# Chemical composition, antioxidant, antimicrobial and cytotoxic activities of *Tagetes minuta* and *Ocimum basilicum* essential oils

**DOI:** 10.1002/fsn3.85

**Published:** 2014-01-16

**Authors:** Mohsen Taheri Shirazi, Hamid Gholami, Gholamreza Kavoosi, Vahid Rowshan, Asad Tafsiry

**Affiliations:** 1 Institute of Biotechnology, Shiraz UniversityShiraz, 71441-65186, Iran; 2 Department of Natural Resources, Fars Research center for Agriculture and Natural ResourcesShiraz, 19395-3697, Iran

**Keywords:** Antimicrobial, cytotoxic, *Ocimum basilicum*, radical scavenging, *Tagetes minuta*

## Abstract

Chemical composition, antioxidant, antimicrobial and cytotoxic activities of *Tagetes minuta* (TM) essential oil (TMO) and *Ocimum basilicum* (OB) essential oil (OBO) were examined. The main components for TMO were dihydrotagetone (33.9%), E-ocimene (19.9%), tagetone (16.1%), *cis*-*β*-ocimene (7.9%), Z-ocimene (5.3%), limonene (3.1%) and epoxyocimene (2.03%). The main components for OBO were methylchavicol (46.9%), geranial (19.1%), neral (15.15%), geraniol (3.0%), nerol (3.0%), caryophyllene (2.4%). Inhibitory concentrations (IC_50_) for reactive oxygen species (ROS) and reactive nitrogen species (RNS) scavenging were 12–17 and 200–250 *μ*g/mL of TMO and OBO, respectively. Minimal inhibitory concentration (MIC) against *Salmonella typhi*,*Escherichia coli*,*Staphylococcus aureus*,*Bacillus subtilis*,*Aspergillus niger*, and *Candida albicans* were 150 ± 8, 165 ± 9, 67 ± 8, 75 ± 7, 135 ± 15, and 115 ± 8 *μ*g/mL of TMO, respectively. MIC for *S. typhi*,*E. coli*,*S. aureus*,*B. subtilis*,*A. niger*, and *C. albicans* were 145 ± 8, 160 ± 7, 45 ± 4, 40 ± 3, 80 ± 9, and 95 ± 7 *μ*g/mL of OBO, respectively. IC_50_ for nasopharyngeal cancer cell line (KB) and liver hepatocellular carcinoma cell line (HepG2) were 75 ± 5 and 70 ± 4 *μ*g/mL of TMO, respectively. IC_50_ for KB and HepG2 were 45 ± 4 and 40 ± 3 *μ*g/mL of OBO, respectively. Thus, they could be used as an effective source of natural antioxidant and antibacterial additive to protect foods from oxidative damages and foodborne pathogens. Furthermore, they could be promising candidate for antitumor drug design.

## Introduction

*Tagetes minuta* (TM) is a marigold plant in the sunflower (Asteraceae) family. *Tagetes* species originally has been used as a source of essential oil for the flavoring in the food industries. The powders and extracts of *Tagetes* are rich in the orange-yellow carotenoid and are used as a food colorant in foods such as pasta, vegetable oil, margarine, mayonnaises, salad dressing, baked goods, confectionery, dairy products, ice cream, yogurt, citrus juice, mustard and as colorant in poultry feed (Iranian Herbal Pharmacopoeia 2002; Zhang et al. 2009; Nerio et al. 2010). TM is also extensively used medicinally as a condiment and herbal tea in a wide variety of fields in its native region and as a popular traditional folk remedies or in the complementary and medical therapy. TM has several medical benefits such as remedy for colds, respiratory inflammations, stomach problem, anti-spasmodic, anti-parasitic, anti-septic, insecticide, and sedative. It is used for chest infections, coughs and catarrh, dilating the bronchi, facilitating the flow of mucus and dislodging congestion and can be used in cases of skin infections. It also has a healing effect on wounds, cuts, calluses and bunions (Gillij et al. 2008; Rahimi et al. 2010; Maity et al. 2011).

*Ocimium basilicum* (OB) is a culinary plant belonging to the Lamiaceae family that is extensively used as a flavoring agent in a wide variety of fields in its native region and as a popular traditional folk remedies or in complementary and alternative medical therapy. This plant has several functional characteristics including carminative, stimulant, diaphoretic, diuretic, dyspepsia, antiseptic, anesthetic, flatulence, gastritis, anti-spasmodic, anthelmintic, anti-diarrheal, analgesic and anti-tussive. Other medicinal uses of OB include treatment of some gastrointestinal disorders, gastrodynia, diarrhea and vomiting (Mondal et al. 2009; Nerio et al. 2010; Rahimi et al. 2010). Considering the aforementioned pharmacological and therapeutic properties, OB has played an important role not only in traditional medicine but also in modern pharmacological and clinical investigations. In this perspective, the OB essential oil (OBO) has played a crucial role in pharmaceutical as well as food industries. However, such practices are largely based on folklore and train of traditional medicine rather than evidence-based research.

In the present study, reactive oxygen species (ROS) and reactive nitrogen species (RNS) scavenging activities of the TMO and OBO were examined using 2, 2′-azino-di (3-ethylbenzthiazoline-6-sulfonate) (ABTS) and sodium nitrite scavenging effects, respectively. The TMO and OBO were individually tested against two Gram-negative bacteria (*Salmonella typhi* and *Escherichia coli*), two Gram-positive bacteria (*Staphylococcus aureus* and *Bacillus subtilis*) and two fungi (*Aspergillus niger and Candida albicans*) caused foodborne outbreaks and illnesses. Cytotoxic activity of TMO and OBO against nasopharyngeal cancer cell line (KB) and liver hepatocellular carcinoma cell line (HepG2) was examined using a modified 3-(4, 5-dimethylthiazol-2-yl)-2, 5-diphenyltetrazolium bromide test (MTT) assay. In this study, we offer that TMO and OBO has radical scavenging, antimicrobial and cytotoxic activities and could be used as safe and effective source of natural antioxidant to improve the oxidative stability of fatty foods during storage and also can be used as a safe antimicrobial additive to protect foods from foodborne pathogens. Furthermore, they can be used as candidate for antitumor drug design.

## Materials and Methods

### Plant materials and isolation of the essential oils

Seed of TM was obtained from Institute of Medicinal Plants, Isfahan, Iran and was grown in green house conditions in Sadra near Shiraz, Iran. The seeds of TM were sown in experimental greenhouse, in September 2011. One month later obtained seedlings were transferred to experimental field and distributed homogenously. The aerial parts of plants were harvested at the flowering stage. The leaves of the plants were separated from the stem and were dried in the shade for 72 h. Seeds of a native OB plants were directly sowed in experimental field. During the germination period and first 2 weeks of plantlet growth, they were irrigated with tap water. The aerial parts of plants were harvested at the flowering stage, dissected to leaves and dried at room temperature for 4–5 days. The shade dried leaves (100 g) were hydro-distilled for 3 h using an all-glass Clevenger-type apparatus (Herbal Exir Co., Mashhad, Iran) according to the method outlined by the British Pharmacopeia (1998). The TMO and OBO were dehydrated over anhydrous sodium sulfate and stored at −20°C until analyzed by gas chromatography–mass spectrometry (GC-MS) and then used for biological assays.

### Identification of the essential oils components

The essential oils analysis was performed using an Agilent gas chromatograph series 7890-A (Agilent, Palo Alto, CA) with a flame ionization detector (FID). The analysis was carried out on fused silica capillary HP-5 column (30 m × 0.32 mm i.d.; film thickness 0.25 *μ*m). The injector and detector temperatures were kept at 250 and 280°C, respectively. Nitrogen was used as carrier gas at a flow rate of 1 mL/min; oven temperature program was 60–210°C at the rate of 4°C/min and then programmed to 240°C at the rate of 20°C/min and finally held isothermally for 8.5 min; split ratio was 1:50. GC–MS analysis was carried out by use of Agilent gas chromatograph equipped with fused silica capillary HP-5MS column (30 m × 0.25 mm i.d.; film thickness 0.25 *μ*m) coupled with 5975-C mass spectrometer. Helium was used as carrier gas with ionization voltage of 70 eV. Ion source and interface temperatures were 230 and 280°C, respectively. Mass range was from 45 to 550 amu. Oven temperature program was the same given above for the GC. Retention indices (RI) were determined using retention times (RT) of n-alkanes (C_8_-C_25_) that were injected after the essential oil under the same chromatographic conditions. The retention indices for all components were determined according to the method of using n-alkanes as standard(s). The compounds were identified by comparison of retention indices (RI, HP-5) with those reported in the literature and by comparison of their mass spectra with the Wiley GC/MS Library and Mass Finder 2.1 Library data published mass spectra data (Adams 2007).

### ROS scavenging assay of essential oils

To assay ROS scavenging activity, 10 *μ*L of the essential oils (0–500 *μ*g/mL in DMSO) was added to 1.0 mL of diluted ABTS radical solution (7 mmol/L ABTS and 2.54 mmol/L potassium persulfate). After mixing, the absorbance (A) was read at 734 nm against a blank solution containing 1% DMSO using an Ultrospec 2000 spectrophotometer (Pharmacia, Uppsala, Sweden). The percentage of ROS scavenging was calculated as: ([A734_blank_ − A734_sample_]/A734_blank_) × 100. The concentrations that could provide 50% inhibition (IC_50_) were calculated from the graph that plotted the inhibition percentage against different essential oil concentrations (Kavoosi and Rowshan 2013).

### RNS scavenging assay of essential oils

To assay RNS scavenging activity, 10 *μ*L of the essential oils (0–500 *μ*g/mL in DMSO) was incubated with 0.5 mL of sodium nitrite (10 *μ*g/mL in 100 mmol/L sodium citrate pH 5) at 37°C for 2 h. After incubation, 0.5 mL of Griess reagent was added and the absorbance (A) was read at 540 nm using a spectrophotometer. A solution containing 1% DMSO without essential oil was used as blank. The percentage of RNS scavenging was calculated as follows: ([A540_blank_ − A540_sample_]/A540_blank_) × 100. IC_50_ was calculated from the graph that plotted the inhibition percentage against different essential oil concentrations (Kavoosi and Rowshan 2013).

### Antibacterial and antifungal activity assay of essential oils

All microorganisms were obtained from the Persian type culture collection (PTCC), Tehran, Iran. The essential oils were individually tested against two Gram-negative bacteria (*S. typhi* PTCC 1609 [Iran isolate] and *E. coli* PTCC 1330 [ATCC 8739]), two Gram-positive bacteria (*S. aureus* PTCC 1112 [ATCC 6538] and *B. subtilis* PTCC 1023 [ATCC 6633]), and two fungi (*A. niger* PTCC 5010 [ATCC 9142] and *C. albicans* PTCC 5027 [ATCC 10231]). Minimum inhibitory concentration (MIC) was determined against serial dilutions of the essential oils (0-200 *μ*g/mL) using microdilution method recommended by Clinical and Laboratory Standards Institute (CLSI) (2006). Bacteria and fungi strains were suspended in Luria-Bertani (LB) media and the densities were adjusted to 0.5 McFarland standards at 640 nm (108 CFU/mL) and then diluted to 105 CFU/mL with LB. Bacteria and fungi suspensions (0.5 mL) and the essential oils (0.5 mL) were added to 1.5 mL microtube and incubated with shaking at 37°C for 24 h. Medium without bacteria and fungi was used as sterility control. Medium with bacteria but without essential oils was used as growth control (blank). Positive control included Gentamicin, Ampicillin and Ketoconazole (all Padtan Teb, Iran, 10 *μ*g/mL) for Gram-negative bacteria, Gram-positive bacteria and fungi, respectively. The growth inhibition was estimated by measuring turbidity of the cultured medium at 640 nm using a spectrophotometer (Pharmacia, Uppsala, Sweden). The percentage of growth inhibition was obtained by the equation: ([A640_blank_ − A640_sample_]/A640_blank_) × 100. MIC was defined as the lowest concentration of the essential oils produced >90% growth reduction compared with the growth in the control microtube. MIC was calculated from a graph that plotted inhibition percentage against different essential oils concentrations.

### Cytotoxic activity of essential oils

The in vitro cytotoxic activity of the essential oils on two human tumor cell lines, viz, nasopharyngeal cancer (KB) and liver hepatocellular carcinoma (HepG2) cell lines were examined using a modified MTT assay (Nouri et al. 2000). All cell lines were obtained from the cell bank of the Pasteur Institute of Iran. The cells were cultured in a humidified atmosphere at 37°C using RPMI-1640 (Gibco, Karlsruhe, Germany) supplemented with 10% fetal bovine serum (Gibco), 100 U/mL penicillin (Sigma-Aldrich, Saint Louis, MO) and 100 *μ*g/mL streptomycin (Sigma-Aldrich) in a 5% CO_2_ incubator. The MTT assay was carried out as follows. Briefly, 180 *μ*L of medium containing cells at a density of 2 × 10^4^ cells/mL were seeded in each well of a flat-bottom 96-well plate (Tissue Culture Plate, Jet Biofil, Kyoto, Japan). Cells were permitted to adhere to the plate for 24 h. The adhered cells were treated with various concentrations of the essential oils (0–100 *μ*g/mL in DMSO) and incubated for 24 h. Finally, the medium was replaced with 200 *μ*L fresh medium containing 0.5 mg/mL of MTT (Sigma-Aldrich). Plates were incubated at 37°C for another 4 h after which the medium was discarded and formazan blue, which was formed in the cells, was dissolved with 100 *μ*L DMSO at 37°C for 10 min. All tests and analyses were run in triplicate. DMSO was used as the control. The absorbance of each well was determined by spectrophotometer at dual wavelengths of 570 and 630 nm on a micro plate ELISA reader (BioTek Elx 808, Winooski, VT). Viability percentage was calculated by the following formula: (absorbance of treated cells/absorbance of corresponding control) × 100. The control was essential oil-untreated cells containing DMSO at the highest concentration used (1%). The concentration providing 50% inhibition (IC_50_) was calculated from a graph plotting inhibition percentage against different essential oil concentrations.

### Statistical analysis

All data are expressed as the means ± standard deviations of at least three independent experiments. The significant differences between treatments were analyzed by one-way analysis of variance (ANOVA) test at *P* < 0.01 using statistical package for the social sciences (SPSS, Abaus Concepts, Berkeley, CA) and Prism 5 (Graph Phad, San Diego, CA) software.

## Results

### Chemical composition of TMO and OBO

GC-MS analysis of the TMO indicated that the main components were dihydrotagetone (33.9%), E-ocimene (19.9%), tagetone (16.1%), *cis*-*β*-ocimene (7.9%), Z-ocimene (5.3%), limonene (3.1%), and epoxyocimene (2.0%) (Table 1). GC-MS analysis of the OBO indicated that the main components were methylchavicol (46.9%), geranial (19.1%), neral (15.1%), geraniol (3.0%), nerol (2.9%), caryophyllene (2.4%) (Table 2).

**Table 1:** Chemical composition of essential oil from *Tagetes minuta*.

Compounds	RI^1^	% of compounds
*α*-Pinene	933	0.33
Sabinene	973	0.40
*cis*-3-Hexenyl acetate	1005	0.15
*p*-Cymene	1025	0.93
Limonene	1029	3.10
*cis*-*β*-Ocimene	1037	7.90
Dihydrotagetone	1061	33.8
allo-Ocimene	1135	0.36
E, Z-Epoxyocimene	1149	2.00
Tagetone	1160	16.1
*cis*-Tagetone	1167	0.20
*p*-Mentha-1,8 dien-3-one	1208	0.18
Z-Ocimene	1238	5.30
E-Ocimene	1250	19.9
Thymol	1282	0.48
Carvacrol	1298	0.47
*cis*-Isoeugenol	1398	0.86
E-caryophyllene	1420	0.32
*α*-Humulene	1455	0.15
Germocrene D	1497	0.43
Spathulenol	1582	0.42

1 RI, retention indices relative to C8–C25 n-alkanes on the HP-5 column.

**Table 2:** Chemical composition of essential oil from *Ocimum basilicum*.

Compounds	RI^1^	% of compounds
*α*-Pinene	933	0.3
1-Octen-3-ol	980	0.1
5-Hepten-2-one-6-methyl	991	1.0
*p*-Cymene	1024	0.1
Limonene	1028	0.2
*trans*-*β*-Ocimene	1046	0.3
*cis*-Sabinene hydrate	1073	0.2
*cis*-Verbenol	1142	0.2
Borneol	1153	0.5
*p*-Mentha 1,5-dien-8 ol	1166	0.4
Methylchavicol	1211	47
*n*-Octyl acetate	1215	0.4
Nerol	1238	3.2
Neral	1253	15
Geraniol	1265	3.1
Geranial	1284	19
Neryl acetate	1367	0.3
Geranyl acetate	1387	0.3
Methyl eugenol	1409	0.5
E-Caryophyllene	1422	2.4
*trans*-*α*-Bergamotene	1436	0.5
*α*-Humulene	1456	1.1
Germacrene D	1483	1.2
*trans*-*α*-Bisabolene	1543	1.6
Caryophyllene oxide	1589	0.4

1 RI, retention indices relative to C8–C25 n-alkanes on the HP-5 column.

### Antioxidant activity of TMO and OBO

Both TMO and OBO displayed a concentration dependent ROS and RNS scavenging activity (Table 3). Inhibitory concentration (IC_50_) for ROS and RNS scavenging were 12 ± 2 and 13 ± 4 *μ*g/mL of TMO, respectively. IC_50_ for ROS and RNS scavenging were 200 ± 11 and 210 ± 16 *μ*g/mL of OBO, respectively. TMO had stronger radical scavenging capacity than OBO.

**Table 3:** Radical scavenging activity of essential oils from *Tagetes minuta* and *Ocimum basilicum*.

IC_50_ (*μ*g/mL) for:	*T. minuta*	*O. basilicum*
ROS	12 ± 2	200 ± 11
RNS	13 ± 4	210 ± 16

IC_50_, effective concentration of the test compound which scavenge the radical by 50%. IC, inhibitory concentration; ROS, reactive oxygen species; RNS, reactive nitrogen species.

### Antibacterial activity of TMO and OBO

Both TMO and OBO displayed a concentration-dependent antibacterial activity (Table 4). MIC for *S. typhi*,*E. coli*,*S. aureus*,*and B. subtiliss* were 150 ± 8, 165 ± 9, 67 ± 8, and 75 ± 7 *μ*g/mL of TMO, respectively. MIC for *S. typhi*,*E. coli*,*S. aureus*, and *B. subtilis* were 145 ± 8, 160 ± 7, 45 ± 4, and 40 ± 3 *μ*g/mL of OBO, respectively. TMO and OBO had stronger effects on the Gram-positive bacteria than that of Gram-negative bacteria.

**Table 4:** Antibacterial activity of *Tagetes minuta* and *Ocimum basilicum* essential oils.

MIC (*μ*g/mL) for:	*T. minuta*	*O. basilicum*
*Salmonella typhi*	150 ± 8	145 ± 8
*Escherichia coli*	165 ± 9	160 ± 7
*Staphylococcus aureus*	67 ± 8	45 ± 4
*Bacillus subtilis*	75 ± 7	40 ± 3

Data are expressed as means ± standard deviation. MIC, minimum inhibitory concentration. MIC was defined as the lowest concentration of the compounds produced >90% growth reduction compared with the growth in the control well.

### Antifungal activity of TMO and OBO

TMO and OBO displayed a concentration-dependent antifungal activity (Table 5). MIC for *A. niger* and *C. albicans* were 135 ± 15, and 115 ± 8 *μ*g/mL of TMO, respectively. MIC for *A. niger* and *C. albicans* were 80 ± 9, and 95 ± 7 *μ*g/mL of OBO, respectively.

**Table 5:** Antifungal activity of *Tagetes minuta* and *Ocimum basilicum* essential oils.

MIC (*μ*g/mL) for:	*T. minuta*	*O. basilicum*
*Aspergillus niger*	135 ± 15	80 ± 9
*Candida albicans*	115 ± 8	95 ± 7

Data are expressed as means ± standard deviation. MIC, minimum inhibitory concentration. MIC was defined as the lowest concentration of the compounds produced >90% growth reduction compared with the growth in the control well.

### Cytotoxic activity of TMO and OBO

The MTT assay results indicated that at low concentrations (<50 *μ*g/mL), TMO had no effect on KB and HepG2 viability. However, at higher concentrations (50–200 *μ*g/mL), cell viability was significantly reduced in a concentration-related manner, with the maximum effect at concentrations >200 *μ*g/mL. IC_50_ for KB and HepG2 was 75 ± 5 and 70 ± 4 *μ*g/mL of TMO, respectively (Table 6). The MTT assay results indicated that low concentrations (1–10 *μ*g/mL), OBO had no effect on KB and HepG2 viability. However, at higher concentrations (10–100 *μ*g/mL), cell viability was significantly reduced in a concentration-related manner, with the maximum effect at 200 *μ*g/mL. IC_50_ for KB and HepG2 was 45 ± 4 and 40 ± 3 *μ*g/mL of OBO, respectively (Table 6).

**Table 6:** Cytotoxic activity of *Tagetes minuta* and *Ocimum basilicum* essential oils.

IC_50_ (*μ*g/mL) for:	*T. minuta*	*O. basilicum*
KB	75 ± 5	45 ± 4
HepG-2	70 ± 4	40 ± 3

Data are expressed as the means ± standard deviations for at least three independent experiments. IC_50_, effective concentration of the test compound which kills 50% of the cells tested; KB, human nasopharyngeal cancer cell line; HepG2, liver hepatocellular carcinoma cell line.

## Discussions

GC-MS analysis of the essential oil indicated the main components TMO were dihydrotagetone, E-ocimene, tagetone, *cis*-*β*-ocimene, Z-ocimene, limonene and epoxyocimene. Previous study reported the main components of TMO were *β*-ocimene, dihydrotagetone, tagetone, Z-ocimene and E-ocimene (Lopez et al. 2011). Another study reported thiophenes and polyacetylenic compounds in the *Tagetes* species and TM had the highest total thiophene yield (Marotti et al. 2010). Accordingly, the main components of TMO could be tagetone (*cis*/*trans*, ketone/alcohol, aldehyde/alcohol), ocimene (*cis*/*trans*, ketone/alcohol, aldehyde/alcohol) and thiophene derivatives (Breme et al. 2009; Marotti et al. 2010; Ranilla et al. 2010; Garcia et al. 2011; Lopez et al. 2011; Armas et al. 2012). Analysis of the chemical composition of the OBO by GC-MS indicated that methylchavicol, geranial, neral, geraniol, nerol and caryophyllene were the main constituents. According to previous studies, the most abundant components in OBO are phenol-containing monoterpenes (methylchavicol), alcoholic monoterpenes (linalool, geraniol, nerol), acyclic monoterpene aldehyde (geranial, neral), cyclic ether monoterpenes (1, 8-cineole) and carbure bicyclic sesquiterpene (caryophyllene) (Rao et al. 2011; Venancio et al. 2011; Verma et al. 2012). Therefore, the plants analyzed in this research had roughly same components with other previously analyzed essential oils, however, showed important differences in their quality and quantity of components.

The TMO analyzed here possessed potent in vitro ROS and RNS scavenging activity. The TMO at concentration >30 *μ*g/mL had the ability to scavenge all ROS and RNS radicals. ROS are oxygen-derived small molecules, including oxygen radicals such as superoxide, hydroxyl and peroxyl and some non-radicals that are easily converted into radicals, such as hydrogen peroxide. ROS, once produced, can interact with various molecules including other small inorganic molecules as well as macromolecules such as proteins and lipids. During these interactions, ROS may destroy or change the function of the target molecule (Circu and Aw 2010). The ROS reducing activity of TMO observed in our study implies the beneficial role of this product for reducing damages in biological tissues. The radical scavenging activity of compounds is mainly due to their oxidation-reduction potential, which can play an important role in neutralizing free radicals. This activity is related to phenolic hydroxyl groups (Katalinic et al. 2006). TMO mainly contains dihydrotagetone, ocimene, tagetone and limonene which all are monoterpenes. Despite polyphenol absence TMO exhibited significant radical scavenging activity. This antioxidant activity was confirmed by previous researches with IC50 between 35 and 344 *μ*g/mL (Gong et al. 2012; Gupta et al. 2012; Perez-Cruz et al. 2013). Thus, TMO analyzed in this research showed stronger antioxidant activity compared to previously analyzed TMOs.

The OBO analyzed in this research showed potent in vitro ROS and RNS scavenging activity. The OBO at concentrations more than 500 *μ*g/mL had the ability to scavenge all ROS and RNS radicals which indicates its moderate radical scavenging activity. The OBO also possessed antioxidant activity as measured by the 2, 2-diphenyl-1-picrylhydrazyl (DPPH) and ABTS assay (Tarchoune et al. 2010; Kaurinovic et al. 2011; Sgherri et al. 2011). OBO mainly contains methylchavicol, geranial/geraniol and neral/nerol. The radical scavenging activity of methylchavicol and essential oil bearing methylchavicol to some extent was confirmed (Tominaga et al. 2005; Mohamad et al. 2011). Previous studies on the radical scavenging activity and antioxidant capacity of geraniol and geraniol bearing essential oil using tsigniertiary-butyl hydroperoxide stressed rat alveolar macrophages clearly showed that geraniol increased the cell viability and showed 45% increase in superoxide dismutase (antioxidant enzyme) activity and 120% increase in glutathione content (antioxidant). Geraniol was found to have radical scavenging and significantly decreased lipid peroxidation and inhibited nitric oxide (NO) release and ROS generation in pretreated cells as compared to stressed cells. These results indicated the antioxidant activity of geraniol by scavenging ROS and RNS and induction of antioxidant defense systems such as superoxide dismutase and glutathione (Choi et al. 2000; Tiwari et al. 2010). Thus, the radical scavenging activity of OBO could be related to geraniol/geranial followed by methylchavicol. The ROS and RNS reducing activities of OBO observed in our study imply the beneficial role of this product for reducing damages in biological tissues.

TMO analyzed in this research also showed potent antibacterial activities. TMO has a significant antibacterial activity against both gram-positive and gram-negative bacteria (Hethelyi et al. 1986; Tereschuk et al. 1997; Cespedes et al. 2006). Gonzalez and Marioli studied antibacterial activity of both water extracts (the water remaining after hydro-distillation) and essential oils of TM against *Paenibacillus larvae*. The water remaining after hydro-distillation showed the highest antibacterial activities while essential oils had less activity for the inhibition of *P. larvae* (Gonzalez and Marioli 2010). The total extract and fractions with different solvents, obtained from leaves of TM showed several degrees of antimicrobial activity against Gram-positive and Gram-negative bacteria. The same fractions were inactive against *Lactobacillus* (Gram-positive), *Zymomonas* (Gram-negative) and *Saccharomyces* (fungi kingdom) species (Tereschuk et al. 1997).

OBO analyzed in this research also showed potent antibacterial activities. OBO is a good source of phenol-containing monoterpenes, with a significant antibacterial activity against *S. aureus*,*Salmonella enteritidis*, and *E. coli* and antiseptic against *Proteus vulgaris*,*B. subtilis* and *Salmonella parathypha*. The essential oil of sweet basil oil displayed a great potential for antibacterial activity with their MIC values of 62.5–500 *μ*g/mL, which is similar to our results (Hossain et al. 2010; Rattanachaikunsopon and Phumkhachorn 2010). Previously identified major compounds of OBO are methylchavicol, gitoxigenin, trimethoquinol, *β*-guaiene, aciphyllene, alizarin, naphthaline, caryophyllene and mequinol (Hossain et al. 2010). Considering high concentration of methylchavicol (78%) in essential oil of *O. basilicum* and showing broad spectrum antibacterial and antifungal activities *(*Rao et al. 2011), our results, as we expected, exhibited high antimicrobial activities.

The antibacterial activity which is recognized in the essential oils of several medicinal plants established that the antibacterial activity of essential oils are related to the attack on the phospholipids present in the cell membranes, which causes increased permeability and leakage of cytoplasm, or to their interaction with enzymes located on the cell wall (Paparella et al. 2008). Thus, the resistance of Gram-negative bacteria to the essential oils likely laid in the protective role of their cell wall lipopolysaccharide or outer membranes proteins, which restricts diffusion of hydrophobic compounds through its lipopolysaccharide layer (Oussalah et al. 2007; Garcia-Garcia et al. 2011). Essential oils have the ability to disrupt lipid structure of the cell wall of bacteria, leading to destruction of cell membrane, cytoplasmic leakage, cell lysis and ultimately cell death. The decrease in pH that occurs due to cell membrane disruption results in a loss of control of cellular process such as ATP biosynthesis, DNA transcription and protein synthesis (Xu et al. 2008).

TMO analyzed in this research also showed antifungal activities. Several studies have also described antifungal activities of TMO against Candida, Penicillium and Aspergillus species (Dunkel et al. 2010; Thembo et al. 2010). The antifungal activity of aqueous and organic extracts of *T. minuta*,*Lippia javanica*,*Amaranthus spinosus* and *Vigna unguiculata* against *Fusarium verticillioides*,*F. proliferatum*,*Aspergillus flavus* and *A. parasiticus* were investigated. All extracts except for the water extracts showed growth inhibitory activity against most isolates of the Fusarium spp. The most active were the methanol and hexane extracts of *V. unguiculata* and *A. spinosus* with MIC values of 500 *μ*g/mL against Fusarium spp. (Thembo et al. 2010). These antifungal activities may be attributed, at least in part, to the presence of thiophenes and flavonoids in the extracts (Gonzalez and Marioli 2010; Thembo et al. 2010).

OBO analyzed in this research also showed antifungal activities. Several studies have also described antifungal activities of OBO against *C. albicans*,*Penicillium natum, A. niger* and *Microsporum gyseum* (Siddiqui et al. 2012). Essential oils from *O. basilicum* (methylchavicol 78%), *O. gratissimum* (eugenol 84%), *O. tenuiflorum* (methyeugenol 72%) and *O. kilimandscharicum* (camphor 51.7%) were exhibited broad spectrum antifungal activities (Rao et al. 2011). The effects of essential oils from sweet *O. basilicum* (linalool 65%) on fungal decay and quality parameters of the Thompson seedless table grape were evaluated. Results showed that the essential oils have a good inhibitory effect on the development of fungal decay in Thompson table grapes (Abdollahi et al. 2012). The essential oil of the aerial parts of *O. basilicum* mainly constituted from linalool (29.68%), (Z)-cinnamic acid methyl ester (21.49%), cyclohexene (4.41%) and showed significant antifungal activity against some plant pathogenic fungi (Zhang et al. 2009).

Essential oils and their components generally displayed potent fungicidal activity and flow cytometry confirmed the occurrence of damage to the plasma membrane and cell death (Vale-Silva et al. 2012). In addition, fungicidal activity of essential oil apparently originates from the inhibition of ergosterol biosynthesis and the disruption of membrane integrity (Ahmad et al. 2011). Furthermore, fungicidal activity of essential oils might be due to the induction of calcium stress and up-regulation of genes involved in metabolic and energy pathways, stress response, autophagy, and drug efflux (Rao et al. 2010).

The results of MTT assay indicated that TMO had no effects on the viability of KB and HepG2 cells at low concentrations (<50 *μ*g/mL). However, at higher concentrations (50–200 *μ*g/mL) cell viability was significantly reduced in a concentration dependent manner, the maximum effect was 100% at concentrations >200 *μ*g/mL. Cytotoxic activity of the ethanol extract of *T. eracta* roots against prostate (PC-3) and HeLa cancer cell lines was investigated by Gupta et al. using MTT assay. The extract conferred noticeable cytotoxicity against both PC-3 and HeLa cell lines with IC_50_ of 407 and 164 *μ*g/mL, respectively (Gupta et al. 2012), which are lower rather than TMO analyzed in this research. The results of MTT assay indicated that OBO had no effects on the viability of KB and HepG2 cells at low concentrations (<10 *μ*g/mL). However, at higher concentrations (10–100 *μ*g/mL) cell viability was significantly reduced in a concentration dependent manner, the maximum effect was 100% at 200 *μ*g/mL. The in vitro anticancer activity of the essential oil from *O. basilicum* was examined using methyl thiazol tetrazolium assay against the human cervical cancer cell line (HeLa) and human laryngeal epithelial carcinoma cell line (Hep-2). The IC_50_ values obtained were 90.5 and 96.3 *μ*g/mL, respectively. In other studies the major constituents were found to be methyl cinnamate (70.1%), linalool (17.5%), *β*-elemene (2.6%) and camphor (1.52%) (Kathirvel and Ravi 2012). The major oil constituents of *O. basilicum* were linalool (30–40%) and eugenol (8–30%) had no cytotoxic activity to mammalian cells (Zheljazkov et al. 2008).

Although the constituents of essential oils can act as antioxidants, they may also act as prooxidants and affect inner cell membranes and organelles such as mitochondria in eukaryotic cells. Depending on the type and concentration, this effect may result in cellular cytotoxicity. The essential oils that showing different levels of cytotoxicity also exhibited different antioxidative capacities depending on the composition of the oil especially on their phenolic content (Bakkali et al. 2008). Essential oils by penetrating through the cytoplasmic and organelles membranes disrupt and permeabilize them and especially damage mitochondrial membranes. The mitochondria by changes in electron flow through the electron transport chain produce free radicals which oxidize and damage lipids, proteins and DNA. Phenolic components of essential oils are oxidized by contact with ROS producing very reactive phenoxyl radicals. These types of radical reactions are enhanced by the presence of transition metal ions such as Fe^2+^ and Cu^2+^ (Shamim et al. 2008). Phenolic compounds oxidation appears to take place in the cytosol by contact with ROS to form phenoxyl radical. In the presence O_2_, transition Fe^2+^ and Cu^2+^ metal ions catalyze the oxidation of phenol ring forming phenoxyl radical leading to the formation of ROS and hydroxyl radical then damage mitochondria (Kyselova 2011). Lipophilic phenolic compounds themselves permeabilize the mitochondrial membranes where transition metal ions Fe^2+^ and Cu^2+^ are sequestered in the inter membrane space and provoke a leakage of these ions and ROS from mitochondria (Mehta et al. 2006). Thus, phenolic compounds are oxidized during permeabilization and leakage at the mitochondrial level giving rise to phenoxyl radicals which continue prooxidants chain reactions with proteins and DNA and generate new ROS (Hansen et al. 2006).

Components of natural products especially phenolic components which show antioxidant activity, can be oxidized by ROS and thus generate additional radical species like panoxyl, hydroxyl and superoxide radicals and hydrogen peroxide (El-Agamey et al. 2004). Indeed, antioxidants by interacting with ROS are converted into prooxidants which are able to oxidize lipids, proteins and DNA (Atsumi et al. 2006). Volatile terpenic and phenolic components of essential oils can function as prooxidants by affecting the cellular redox status. This may lead to late apoptosis and/or necrosis including damage to proteins and DNA and overall cytotoxic effects (Bakkali et al. 2008). If the antioxidant concentrations is too weak to permeabilize mitochondrial membranes, conversion into prooxidant may not occur, and the antioxidant would keep its activity. Thus, at low concentrations, antioxidant was not oxidized and could not damage mitochondria. In contrast, at high concentration antioxidant could damage and permeabilize mitochondria, oxidized to prooxidant and could react as prooxidant damaging DNA and proteins (Aydin et al. 2005).

## Conclusions

Considering all of these results, TMO and OBO had ROS and RNS scavenging activities. Radical scavenging activity of TMO was higher than that of OBO. Thus, they can be used as a safe, effective and easily accessible source of natural antioxidants to improve the oxidative stability of fatty foods during storage. In addition, TMO and OBO had antibacterial and antifungal activities. Antibacterial and antifungal activities of OBO were higher than that of TMO. Thus, they could be used as safe antimicrobial agents against food burn pathogens to preserve foods staff against these pathogens. Furthermore, TMO and OBO had cytotoxic activity against KB and HepG2 cell lines. Hence, they can be used as candidate for antitumor drug design.

## References

[b1] Abdollahi A, Hassani A, Ghosta Y, Bernousi I, Meshkatalsadat MH, Shabani R (2012). Evaluation of essential oils for maintaining postharvest quality of Thompson seedless table grape. Nat. Prod. Res.

[b2] Adams RP (2007). Identification of essential oil components by gas chromatography/mass spectrometery.

[b3] Ahmad A, Khan A, Akhtar F, Yousuf S, Xess I, Khan LA (2011). Fungicidal activity of thymol and carvacrol by disrupting ergosterol biosynthesis and membrane integrity against Candida. Eur. J. Clin. Microbiol. Infect. Dis.

[b4] Armas K, Rojas J, Rojas L, Morales A (2012). Comparative study of the chemical composition of essential oils of five *Tagetes* species collected in Venezuela. Nat. Prod. Commun.

[b5] Atsumi T, Tonosaki K, Fujisawa S (2006). Induction of early apoptosis and ROS-generation activity in human gingival fibroblasts (HGF) and human submandibular gland carcinoma (HSG) cells treated with curcumin. Arch. Oral Biol.

[b6] Aydin S, Basaran AA, Basaran N (2005). Modulating effects of thyme and its major ingredients on oxidative DNA damage in human lymphocytes. J. Agric. Food Chem.

[b7] Bakkali F, Averbeck S, Averbeck D, Idaomar M (2008). Biological effects of essential oils-a review. Food Chem. Toxicol.

[b8] Breme K, Tournayre P, Fernandez X, Meierhenrich UJ, Brevard H, Joulain D (2009). Identification of odor impact compounds of *Tagetes minuta* L. essential oil: comparison of two GC-olfactometry methods. J. Agric. Food Chem.

[b9] British Pharmacopeia (1998). British pharmacopeia.

[b10] Cespedes CL, Avila JG, Martínez A, Serrato B, Calderon-Mugica JC, Salgado-Garciglia R (2006). Antifungal and antibacterial activities of Mexican tarragon. J. Agric. Food Chem.

[b11] Choi HS, Song HS, Ukeda H, Sawamura M (2000). Radical-scavenging activities of citrus essential oils and their components: detection using 1, 1-diphenyl-2-picrylhydrazyl. J. Agric. Food Chem.

[b12] Circu ML, Aw TY (2010). Reactive oxygen species, cellular redox systems, and apoptosis. Free Radical Biol. Med.

[b13] Clinical and Laboratory Standards Institute (CLSI) (2006). Methods for dilution antimicrobial susceptibility tests for bacteria that grow aerobically, approved standard.

[b15] Dunkel FV, Jaronski ST, Sedlak CW, Meiler SU, Veo KD (2010). Effects of steam-distilled shoot extract of *Tagetes minuta* (Asterales: Asteraceae) and entomopathogenic fungi on larval *Tetanops myopaeformis*. Environ. Entomol.

[b16] El-Agamey A, Lowe GM, McGarvey DJ, Mortensen A, Phillip DM, Truscott TG (2004). Carotenoid radical chemistry and antioxidant/pro-oxidant properties. Arch. Biochem. Biophys.

[b17] Garcia MV, Matias J, Barros JC, Lopes DP, De Lima RS, Andreotti R (2011). Chemical identification of *T. minuta* Linnaeus (Asteraceae) essential oil and its acaricidal effect on ticks. Rev. Bras. Parasitol. Vet.

[b18] Garcia-Garcia R, Lopez-Malo A, Palou E (2011). Bactericidal action of binary and ternary mixtures of carvacrol, thymol, and eugenol against *Listeria innocua*. J. Food Sci.

[b19] Gillij YG, Gleiser RM, Zygadlo JA (2008). Mosquito repellent activity of essential oils of aromatic plants growing in Argentina. Bioresour. Technol.

[b20] Gong Y, Liu X, He WH, Xu HG, Yuan F, Gao YX (2012). Investigation into the antioxidant activity and chemical composition of alcoholic extracts from defatted marigold residue. Fitoterapia.

[b500] Gonzalez MJ, Marioli JM (2010). Antibacterial activity of water extracts and essential oils of various aromatic plants against Paenibacillus larvae, the causative agent of American Foulbrood. J. Invertebr. Pathol.

[b22] Gupta P, Gupta A, Agarwal K, Tomar P, Satija S (2012). Antioxidant and cytotoxic potential of a new thienyl derivative from *T. erecta* roots. Pharm. Biol.

[b23] Hansen JM, Go YM, Jones DP (2006). Nuclear and mitochondrial compartmentation of oxidative stress and redox signaling. Annu. Rev. Pharmacol. Toxicol.

[b501] Hethelyi E, Danos B, Tetenyi P (1986). GC-MS analysis of the essential oils of four Tagetes species and the anti-microbial activity of *T. minuta*. Flav. Fragr. J.

[b24] Hossain MA, Kabir MJ, Salehuddin SM, Rahman SM, Das AK, Singha SK (2010). Antibacterial properties of essential oils and methanol extracts of sweet basil *O. basilicum* occurring in Bangladesh. Pharm. Biol.

[b25] Iranian Herbal Pharmacopoeia (2002).

[b26] Katalinic V, Milos M, Kulisic T, Jukic M (2006). Screening of 70 medicinal plants extracts for antioxidant capacity and total phenols. Food Chem.

[b27] Kathirvel P, Ravi S (2012). Chemical composition of the essential oil from basil (*O. basilicum* Linn.) and its in vitro cytotoxicity against HeLa and HEp-2 human cancer cell lines and NIH 3T3 mouse embryonic fibroblasts. Nat. Prod. Res.

[b28] Kaurinovic B, Popovic M, Vlaisavljevic S, Trivic S (2011). Antioxidant capacity of *O. basilicum* L. and *Origanum vulgare* L. extracts. Molecules.

[b29] Kavoosi G, Rowshan V (2013). Chemical composition, antioxidant and antimicrobial activities of essential oil obtained from Ferula assa-foetida oleo-gum-resin: effect of collection time. Food Chem.

[b30] Kyselova Z (2011). Toxicological aspects of the use of phenolic compounds in disease prevention. Interdiscip. Toxicol.

[b31] Lopez SB, Lopez ML, Aragon LM, Tereschuk ML, Slanis AC, Feresin GE (2011). Composition and anti-insect activity of essential oils from *Tagetes* L. species (*Asteraceae**Helenieae*) on *Ceratitis capitata* Wiedemann and *Triatoma infestans* Klug. J. Agric. Food Chem.

[b32] Maity N, Nema NK, Abedy MK, Sarkar BK, Mukherjee PK (2011). Exploring *T. erecta* Linn flower for the elastase, hyaluronidase and MMP-1 inhibitory activity. J. Ethnopharmacol.

[b33] Marotti I, Marotti M, Piccaglia R, Nastri A, Grandi S, Dinelli G (2010). Thiophene occurrence in different *Tagetes* species: agricultural biomasses as sources of biocidal substances. J. Sci. Food Agric.

[b34] Mehta R, Templeton DM, O'Brien PJ (2006). Mitochondrial involvement in genetically determined transition metal toxicity: II. Copper toxicity. Chem. Biol. Interact.

[b35] Mohamad RH, El-Bastawesy AM, Abdel-Monem MG, Noor AM, Al-Mehdar HA, Sharawy SM (2011). Antioxidant and anticarcinogenic effects of methanolic extract and volatile oil of fennel seeds (*F. vulgare*. J. Med. Food.

[b36] Mondal S, Mirdha BR, Mahapatra SC (2009). The science behind sacredness of Tulsi (*O. sanctum* Linn.). Indian J. Physiol. Pharmacol.

[b37] Nerio LS, Olivero-Verbel J, Stashenko E (2010). Repellent activity of essential oils: a review. Bioresour. Technol.

[b38] Nouri AM, Thompson C, Cannell H, Symes M, Purkiss S, Amirghofran Z (2000). Profile of epidermal growth factor receptor (EGFr) expression in human malignancies: effects of exposure to EGF and its biological influence on established human tumor cell lines. Int. J. Mol. Med.

[b39] Oussalah M, CailleT S, Saucier L, Lacroix M (2007). Inhibitory effects of selected plant essential oils on the growth of four pathogenic bacteria: *E. coli* O157:H7, *S. typhimurium**S. aureus* and *L. monocytogenes*. Food Control.

[b40] Paparella A, Taccogna L, AguzzI I, Chaves-Lopez C, Serio A, MarsiliO F (2008). Flow cytometric assessment of the antimicrobial activity of essential oils against *L. monocytogenes*. Food Control.

[b41] Perez-Cruz F, Cortes C, Atala E, Bohle P, Valenzuela F, Olea-Azar C (2013). Use of pyrogallol red and pyranine as probes to evaluate antioxidant capacities towards hypochlorite. Molecules.

[b42] Rahimi R, Shams-Ardekani MR, Abdollahi M (2010). A review of the efficacy of traditional Iranian medicine for inflammatory bowel disease. World J. Gastroenterol.

[b43] Ranilla LG, Kwon YI, Apostolidis E, Shetty K (2010). Phenolic compounds, antioxidant activity and in vitro inhibitory potential against key enzymes relevant for hyperglycemia and hypertension of commonly used medicinal plants, herbs and spices in Latin America. Bioresour. Technol.

[b44] Rao A, Zhang Y, Muend S, Rao R (2010). Mechanism of antifungal activity of terpenoid phenols resembles calcium stress and inhibition of the TOR pathway. Antimicrob. Agents Chemother.

[b45] Rao BR, Kotharia SK, Rajput DK, Patel RP, Darokar MP (2011). Chemical and biological diversity in fourteen selections of four *Ocimum* species. Nat. Prod. Commun.

[b46] Rattanachaikunsopon P, Phumkhachorn P (2010). Antimicrobial activity of basil oil against *Salmonella enteritidis* in vitro and in food. Biosci. Biotechnol. Biochem.

[b47] Sgherri C, Pinzino C, Navari-Izzo F, Izzo R (2011). Contribution of major lipophilic antioxidants to the antioxidant activity of basil extracts: an EPR study. J. Sci. Food Agric.

[b48] Shamim U, Hanif S, Ullah MF, Azmi AS, Bhat SH, Hadi SM (2008). Plant polyphenols mobilize nuclear copper in human peripheral lymphocytes leading to oxidatively generated DNA breakage: implications for an anticancer mechanism. Free Radical Res.

[b49] Siddiqui BS, Bhatti HA, Begum S, Perwaiz S (2012). Evaluation of the antimycobacterium activity of the constituents from *O. basilicum* against *Mycobacterium tuberculosis*. J. Ethnopharmacol.

[b50] Tarchoune I, Sgherri C, Izzo R, Lachaal M, Ouerghi Z, Navari-Izzo F (2010). Antioxidative responses of *O. basilicum* to sodium chloride or sodium sulphate salinization. Plant Physiol. Biochem.

[b503] Tereschuk ML, Riera MV, Castro GR, Abdala LR (1997). Antimicrobial activity of flavonoids from leaves of *T. minuta*. J. Ethnopharmacol.

[b51] Thembo KM, Vismer HF, Nyazema NZ, Gelderblom WC, Katerere DR (2010). Antifungal activity of four weedy plant extracts against selected mycotoxigenic fungi. Appl. Microbiol.

[b52] Tiwari M, Dwivedi UN, Kakkar P (2010). Suppression of oxidative stress and pro-inflammatory mediators by *Cymbopogon citratus* extract in lipopolysaccharide stimulated murine alveolar macrophages. Food Chem. Toxicol.

[b53] Tominaga H, Kobayashi Y, Goto T, Kasemura K, Nomura M (2005). DPPH radical-scavenging effect of several phenylpropanoid compounds and their glycoside derivatives. Yakugaku Zasshi.

[b54] Vale-Silva LA, Silva MJ, Oliveira D, Goncalves MJ, Cavaleiro C, SalgueirO L (2012). Correlation of the chemical composition of essential oils from *O. vulgare* subsp. virens with the in vitro activity against clinical yeast and filamentous fungi. J. Med. Microbiol.

[b55] Venancio AM, Onofre AS, Lira AF, Alves PB, Blank AF, Antoniolli AR (2011). Chemical composition, acute toxicity, and antinociceptive activity of the essential oil of a plant breeding cultivar of basil. Planta Med.

[b56] Verma RS, Padalia RC, Chauhan A (2012). Variation in the volatile terpenoids of two industrially important basil cultivars during plant ontogeny in two different cropping seasons from India. J. Sci. Food Agric.

[b57] Xu J, Zhou F, Ji BP, Pei RS, Xu N (2008). The antibacterial mechanism of carvacrol and thymol against Escherichia coli. Lett. Appl. Microbiol.

[b58] Zhang JW, Li SK, Wu WJ (2009). The main chemical composition and in vitro antifungal activity of the essential oils of *O. basilicum*. Molecules.

[b59] Zheljazkov VD, Cantrell CL, Tekwani B, Khan SI (2008). Content, composition, and bioactivity of the essential oils of three basil genotypes as a function of harvesting. J. Agric. Food Chem.

